# Combined Prebiotic Extract of Mung Bean, Red Bean, and Fennel Improves Intestinal Barrier Integrity in HT-29 Cells and DSS-Induced Colitis via Gut Microbiota Alteration

**DOI:** 10.3390/cimb48010032

**Published:** 2025-12-26

**Authors:** Chul Sang Lee, Woo-Young Jang, Ju-Yeon Kim, Myung-Hyun Lee, Sung-Joon Mo, Yong-Tae Kim, Jae-Jung Shim, Jae-Hwan Lee

**Affiliations:** R&BD Center, hy, Co., Ltd., Giheungdanji-ro 24beon-gil, Giheung-gu, Yongin-si 17086, Republic of Korea; chulsangli@hy.co.kr (C.S.L.); j-doryoung@hy.co.kr (W.-Y.J.); 10003016@hy.co.kr (J.-Y.K.); mhlee12@hy.co.kr (M.-H.L.); stal6000@hy.co.kr (S.-J.M.); jjshim@hy.co.kr (J.-J.S.)

**Keywords:** prebiotics, inflammatory bowel disease, tight junction, anti-inflammation, gut microbiota

## Abstract

Inflammatory bowel disease (IBD) involves chronic inflammation and disruption of the intestinal barrier, often accompanied by alterations in gut microbiota composition. This study examined the protective potential of a prebiotic mixture extract (PME) prepared from *Vigna radiata* (mung bean), *Vigna angularis* (red bean), and *Foeniculum vulgare* (fennel) using the HT-29 cell and colitis animal model. PME exhibited concentration-dependent antioxidant activity, with greater radical-scavenging capacity in the ABTS assay than in the DPPH assay. In LPS-stimulated HT-29 epithelial cells, PME reduced the mRNA expression of inflammation-associated genes (TNF-α, IL-1β, NF-κB) and upregulated tight junction markers (CLDN1 and OCLN), demonstrating its anti-inflammatory and supportive effects on the intestinal barrier. Vitexin, a C-glycosylated flavonoid, was detected in PME and is expected to mediate these protective effects. In a DSS-induced colitis mouse model, PME administration alleviated disease severity by increasing colon length, reducing serum levels of inflammatory cytokines and COX-2/PGE2, and restoring intestinal permeability. Furthermore, PME modulated the gut microbiota by enhancing beneficial bacteria such as *Bifidobacterium* and *Faecalibaculum* while suppressing inflammation-associated taxa, including *Escherichia*, *Bacteroides*, and *Mucispirillum*. These improvements collectively suggest that PME reinforces epithelial barrier integrity and promotes intestinal homeostasis through both anti-inflammatory and microbiota-regulating actions.

## 1. Introduction

Inflammatory bowel disease (IBD) is a group of chronic, relapsing inflammatory disorders in the gastrointestinal tract, primarily encompassing ulcerative colitis (UC) and Crohn’s disease (CD) [1]. The development of IBD is driven by complex interactions among genetic susceptibility, abnormal immune regulation, impaired epithelial barriers, and an imbalanced gut microbiota [2]. UC is characterized by chronic inflammation confined to the colon and rectum, usually starting at the rectum and spreading continuously along the mucosal surface [3]. On the other hand, CD is an inflammatory disorder characterized by transmural inflammation that can affect any segment of the gastrointestinal tract, most commonly the ileum and colon [4]. CD differs from UC by exhibiting non-continuous, transmural inflammatory responses that can affect the entire gastrointestinal tract, sometimes leading to intestinal blockage or the development of abnormal passages between organs [5]. Patients with IBD commonly present with gastrointestinal symptoms such as abdominal pain, persistent diarrhea, rectal bleeding, and weight loss, often accompanied by systemic manifestations like fatigue and low-grade fever [6]. Conventional therapies for IBD include amino salicylates, corticosteroids, and biologics targeting TNF-α or other integrins [7]. Despite their therapeutic efficacy, the long-term use of these drugs can result in substantial side effects, such as immunosuppression, liver toxicity, and an elevated risk of infections and malignancies [4]. These limitations underscore the need for safer, more targeted strategies to manage chronic intestinal inflammation, thereby directing attention to traditional medicinal plants with an improved safety profile.

Prebiotics are defined as non-digestible or low-digestible food ingredients that benefit the host organism by selectively stimulating the growth or activity of one or a limited number of beneficial bacteria in the gut [8]. They are reported to exert their effects primarily by serving as fermentable substrates that enhance microbial metabolism, thereby increasing short-chain fatty acid (SCFA) production and, in turn, strengthening intestinal barrier function [9]. In addition to SCFA, prebiotics can promote intestinal health by enhancing the abundance of beneficial microbes, such as *Bifidobacterium* and *Lactobacillus*, by modulating immune responses and reducing mucosal inflammation [10]. Based on these mechanisms, prebiotics are gaining attention as promising alternative therapeutic agents for IBD, offering a dietary means to restore microbial balance, reinforce barrier function, and attenuate intestinal inflammation.

Recent studies have highlighted the potential of prebiotic sources, including *Vigna radiata* (mung bean), *Vigna angularis* (red bean), and *Foeniculum vulgare* (fennel), to improve intestinal health (Table 1). Previous studies by Xu et al. and Ao et al. reported that mung bean ameliorates DSS-induced colitis in mice by modulating gut microbiota and improving intestinal barrier function [11,12]. Additionally, Yin et al. reported that red bean extract treatment enhanced gut barrier integrity in mice with DSS-induced colitis [13]. Moreover, fennel seed extract has also been reported to improve intestinal epithelial health in a colitis animal model [14]. In addition to improvements in intestinal health, *Vigna radiata* (mung bean) is known for its high nutritional value and antioxidant, anti-inflammatory, and anticancer properties [15]. Also, *Vigna angularis* (red bean) is commonly used in traditional medicine and has been shown to have remarkable antioxidant, anti-inflammatory, and anti-obesity effects [16]. Similarly, *Foeniculum vulgare* (fennel), a well-known digestive aid, exhibits significant anti-inflammatory and antioxidant effects [17].

Notably, several of these plant sources contain vitexin, a naturally occurring C-glycosylated flavone that has been widely reported in legumes and various medicinal herbs [18]. Vitexin has been shown to possess potent antioxidant, anti-inflammatory, and epithelial-protective activities in multiple preclinical studies, including the mitigation of intestinal inflammation and preservation of mucosal barrier integrity [19]. These biological properties suggest that vitexin may be one of the bioactive constituents contributing to the gut-protective effects previously reported for mung bean, red bean, and fennel. Guided by this evidence, we formulated the hypothesis that a blend of these plant-based materials would enhance intestinal barrier function. Therefore, this study aimed to evaluate the intestinal protective effects of a hot-water extract mixture prepared from mung bean, red bean, and fennel in LPS-stimulated HT-29 cells and in a DSS-induced colitis mouse model.

## 2. Materials and Methods

### 2.1. Prebiotic Mixture Extract (PME) Sample Preparation

Dried *Vigna radiata* (mung bean), *Vigna angularis* (red bean), and *Foeniculum vulgare* (fennel) were each weighed at 180 g and then combined to a total of 540 g. The prebiotic mixture was extracted with distilled water at a 1:9 (*w*/*v*) ratio by heating at 95 °C for over 2 h. The extract was filtered using Whatman No. 1 filter paper (Whatman International, Ltd., Leeds, England) and concentrated under reduced pressure using a rotary evaporator. Finally, the concentrated extract was freeze-dried, and the resulting powder was stored at room temperature until further use.

### 2.2. High Performance Liquid Chromatography (HPLC) Based Quantification of Vitexin in Prebiotic Mixture Extract (PME)

The PME powder was homogenized and prepared for chromatographic analysis. Approximately 1 g of the sample was suspended in 50% methanol, vortexed thoroughly, and sonicated for 30 min to enhance analyte extraction. The extract was centrifuged at 15,970× *g* for 10 min, after which the supernatant was collected and filtered through a 0.45 μm PTFE syringe filter (Merck, Darmstadt, Germany). A 50 μg/mL vitexin stock solution (Sigma-Aldrich, St. Louis, MO, USA) was prepared in the same solvent system and serially diluted to obtain a five-point calibration series for quantitative analysis.

HPLC analysis was performed on an Agilent 1260 Infinity II system (Agilent, Santa Clara, CA, USA) equipped with a diode array detector (DAD). Chromatographic separation was carried out on a Nomura Chemical Develosil C30-UG column (250 mm × 4.6 mm i.d., 3 μm) (Hinode-cho, Seto, Japan), maintained at 40 °C. The mobile phase consisted of solvent A (0.1% trifluoroacetic acid in water) and solvent B (0.1% trifluoroacetic acid in acetonitrile). The gradient elution program was set as follows: 92% A/8% B at 5 min; 84% A/16% B at 20 min; 76% A/24% B at 35 min; and 10% A/90% B at 40 min, followed by re-equilibration to the initial composition (92% A/8% B) up to 50 min. The flow rate was 0.8 mL/min, and the injection volume was 10 μL. DAD detection was conducted at 320 nm with a reference wavelength of 400 nm to monitor the principal chromophore of vitexin.

Quantitative determination of vitexin was performed using a five-point calibration curve prepared from serial dilutions of the 50 μg/mL vitexin stock solution, with peak areas plotted against the corresponding concentrations. Linear regression yielded the equation y = 15.717x + 5.7069, with a correlation coefficient of R^2^ = 0.9999, indicating excellent linearity across the calibration range.

### 2.3. Cell Culture

HT-29 human epithelial cells were purchased from the Korean Cell Line Bank (KCLB, Seoul, Republic of Korea). The cells were cultured in plastic cell culture dishes in a cell incubator (37 °C, 5% CO_2_) with media supplemented with RPMI 1640 (Gibco, Thermo Fisher Scientific, Waltham, MA, USA), 10% heat-inactivated fetal bovine serum (HI-FBS) (Gibco, Thermo Fisher Scientific, Waltham, MA, USA), and 1% penicillin–streptomycin (Gibco, Thermo Fisher Scientific, Waltham, MA, USA).

### 2.4. Antioxidant Activity Evaluation

The antioxidant capacity of PME was measured in a dose-dependent manner (4, 8, 16, 32, 62.5, 125, 250, 500, 1000 μg/mL). The radical scavenging activity of the PME was assessed using the 2,2′-azino-bis (3-ethylbenzothiazoline-6-sulfonic acid) (ABTS) (Sigma-Aldrich, St. Louis, MO, USA) assay and the 2,2-diphenyl-1-picrylhydrazyl (DPPH) (Sigma-Aldrich, St. Louis, MO, USA) assay, as previously described by Lee et al. [20]. In the ABTS assay, a solution of ABTS radical cations was prepared by reacting ABTS with potassium persulfate (ABTS-PP) (Sigma-Aldrich, St. Louis, MO, USA), and the absorbance of the mixture was measured at 734 nm. The antioxidant activity was determined by measuring the decrease in absorbance of the ABTS-PP solution, with lower absorbance indicating greater scavenging activity. For the DPPH assay, DPPH solution was prepared in methanol, and the reaction mixture was incubated in the dark for 30 min with the sample extract. The reduction in the purple color of the DPPH radical was monitored by measuring the absorbance at 517 nm. A lower absorbance indicates stronger antioxidant activity, as it reflects greater DPPH radical scavenging by the extract. Ascorbic acid (AC) was used as a positive control for both ABTS and DPPH radical scavenging assays. The scavenging activity was expressed using the following equations.

ABTS radical scavenging activity (%) = [(OD734(control) − OD734(sample))/OD734(control)] × 100.

DPPH radical scavenging activity (%) = [(OD517(control) − OD517(sample))/OD517(control)] × 100.

### 2.5. Cell Viability

The cytotoxicity of the PME of HT-29 cells was measured using the MTT (3-(4,5-dimethylthiazol-2-yl)-2,5-diphenyl tetrazolium bromide) (Sigma-Aldrich, St. Louis, MO, USA) assay. HT-29 cells were seeded in a 96-well cell culture plate at a concentration of 1 × 10^4^ per well and stabilized for 24 h. Then, the cells were treated with PME in a dose-dependent manner (0, 4, 8, 16, 32, 62.5, 125, 250, 500, 1000, 2000 μg/mL) for 24 h. The cells were then treated with 10 μL of MTT solution (5 mg/mL) in each well for 4 h at 37 °C to allow formazan crystals to form. After crystal formation, the MTT solution was removed, and the crystals were dissolved in 200 μL of dimethyl sulfoxide (DMSO) (Sigma-Aldrich, St. Louis, MO, USA). Viable cell counts were performed by measuring the OD at 595 nm using a 96-well plate reader (BioTek^®^ Synergy HT Microplate reader, BioTek, Santa Clara, CA, USA). The viability of HT-29 cells is expressed as the relative percentage based on the absorbance of cells grown under normal conditions without any extra treatments.

Moreover, another set was conducted to confirm the proper lipopolysaccharide dosage. The HT-29 cells were treated with LPS (*Escherichia coli* strain 0111:B4; Sigma-Aldrich, St. Louis, MO, USA) in a dose-dependent manner (0, 1, 3, 5, 10, 20, 50, 100 μg/mL) for 24 h. The procedure was then carried out as described above.

### 2.6. mRNA Extraction and Quantitative Real-Time Polymerase Chain Reaction (qRT-PCR) Analysis of LPS-Induced HT-29 Human Epithelial Cells

HT-29 cells were seeded at a density of 2 × 10^5^ per well in 6-well plates and stabilized in RPMI 1640 culture medium (Gibco, Thermo Fisher Scientific, Waltham, MA, USA). The culture medium was then replaced with serum-free medium, followed by co-treatment with LPS (10 μg/mL) (*Escherichia coli* strain 0111:B4; Sigma-Aldrich, St. Louis, MO, USA) and a PME at various concentrations (50, 100, 250, and 500 μg/mL), excluding the Normal group. Total RNA was extracted from cultured cells using the easy-spin Total RNA Extraction Kit (iNtRON Biotechnology, Seoul, Republic of Korea) according to the manufacturer’s instructions. Then, the cDNA synthesis was conducted at 37 °C for 60 min using the Omniscript Reverse Transcription Kit (Qiagen, Hilden, Germany). The targeted genes were quantified by qRT-PCR using TaqMan Gene Expression Master Mix and TaqMan Gene Expression Assays (Applied Biosystems, Foster City, CA, USA). The data were then analyzed using the QuantStudio 6-Flex Real-time PCR System (Applied Biosystems, Foster City, CA, USA). The targeted genes and the TaqMan probes used in this study are listed in Table 2. The mRNA expression levels of each targeted gene was normalized to the internal standard, GAPDH.

### 2.7. Animal Experimental Protocol

**Figure 1:**
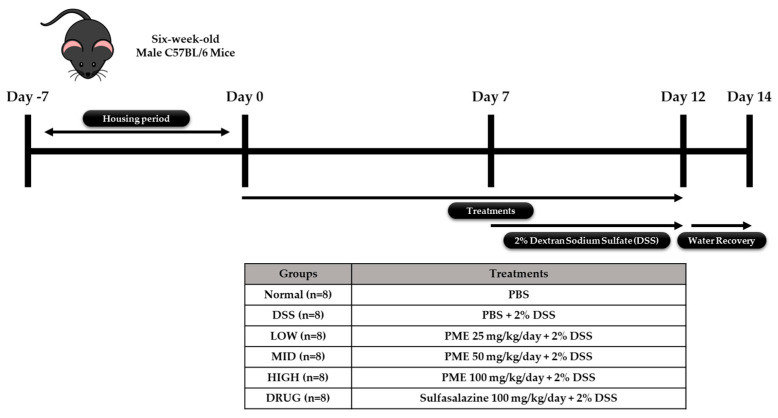
Experimental design of the study.

The animal experiment was performed in accordance with protocols approved by the Ethics Review Committee of R&BD Center, hy Co., Ltd., Republic of Korea (AEC-2025-0004-Y, approval date: 21 July 2025). Forty-eight six-week-old male C57BL/6 mice were purchased from Doo Yeol Biotech (Seoul, Republic of Korea) and were housed at a room temperature of 22 ± 1 °C, with 55 ± 10% humidity and a 12 h light/dark cycle for 1 week. Then, the mice were randomly divided into six groups based on their weight (*n* = 8): Group 1, no treatment (Normal); Group 2, colitis-induced mice (DSS); Group 3, colitis-induced mice + PME 25 mg/kg/day (LOW); Group 4, colitis-induced mice + PME 50 mg/kg/day (MID); Group 5, colitis-induced mice + PME 100 mg/kg/day (HIGH); and Group 6, colitis-induced mice + sulfasalazine (DRUG). After acclimation, mice were pre-treated with sulfasalazine (100 mg/kg/day) and PME (25, 50, 100 mg/kg/day) by oral gavage for 2 weeks. Over the same period, the Normal and DSS groups were orally administered an equal volume of saline. Then, excluding the Normal group, mice were given 2% DSS (MP Biomedicals, Santa Ana, CA, USA; molecular weight 36,000–50,000 Da) in their drinking water for 5 days to induce colitis, followed by a 2-day water recovery period during the second week of treatment. The doses of PME used in the in vivo study (25, 50, and 100 mg/kg) were selected based on preliminary in vitro results. The previous HT-29 study provided insight into the concentrations at which PME exhibited biological activity without causing significant cytotoxicity. The in vitro data were used to define a dose range that would allow for the evaluation of PME’s effects across a spectrum of low to high doses. The chosen doses represent a gradient of LOW (25 mg/kg), MID (50 mg/kg), and HIGH (100 mg/kg) exposure, ensuring a comprehensive assessment of PME’s potential effects. The highest dose of 100 mg/kg was selected to ensure that PME would be administered at a level that was both biologically active and non-toxic for further in vivo assessment. Growth performance data (body weight, feed intake, and water intake) were recorded throughout the study period. The mice were anesthetized in a CO_2_ chamber prior to blood and organ collection at the end of the experimental period. Blood samples were collected and transferred to BD Microtainer^®^ SST^™^ Amber Tubes (Becton, Dickinson and Company, Franklin Lakes, NJ, USA) for serum separation. Liver, ileum, colon, spleen, cecum, and fecal samples were also collected and stored in liquid nitrogen prior to storage at −80 °C for further analysis. The study’s experimental design is shown in Figure 1.

### 2.8. Disease Activity Index (DAI) Score

The DAI scores were performed as described in a previous study with slight modifications [21]. Briefly, body weight loss was graded on a scale of 0 to 6 (0, <0%; 1, 0–5%; 2, 6–10%; 3, 11–15%; 4, 16–20%; 5, 21–25%; and 6, 26–30%) at the endpoint of the experimental period. Intestinal bleeding was also scored from 0 to 3 (0, no blood; 1, slight blood streaks on the colon; 2, moderate visible bleeding on the colon or bedding; and 3, visible severe bleeding) right after sacrifice.

### 2.9. Histological Analysis

The colon length of the mice was measured on a ruler board. Moreover, the distal colon tissues were fixed in 10% formalin solution (Sigma-Aldrich, St. Louis, MO, USA), paraffin-embedded, and stained with hematoxylin and eosin. The stained samples were visualized using a Zeiss Axiovert 200M microscope (Carl Zeiss AG, Thornwood, NY, USA). Histological damage to the colon was quantified by scoring inflammation severity, inflammation extension, and crypt damage as described previously [22]. The inflammation severity was scored on a scale of 0 to 3 (0, none; 1, mild; 2, moderate; 3, severe), and the extent of inflammation was scored from 0 to 3 (0, none; 1, mucosa; 2, mucosa and submucosa; 3, transmural). Crypt damage was scored from 0 to 4 according to the extent of crypt loss and epithelial erosion (0, none; 1, basal 1/3 damaged; 2, basal 2/3 damaged; 3, entire crypts lost with surface epithelium present; 4, crypts and surface epithelium completely lost). Histological analysis of H&E-stained slides and scoring were performed by Doo Yeol Biotech (Seoul, Republic of Korea).

### 2.10. Ileal Permeability

Ileal permeability was determined as previously described [23]. Briefly, dissected ileal segments were ligated at one end and filled with 100 μL of fluorescein isothiocyanate (FITC)-dextran (FD-4; 4 kDa, 40 mg/mL) (Sigma-Aldrich, St. Louis, MO, USA) via intraluminal injection. The opposite end was tied, and the closed segments were placed in modified Krebs–Henseleit bicarbonate buffer (KHBB) (Sigma-Aldrich, St. Louis, MO, USA) (pH 7.4). Samples were incubated at 37 °C for 20 min. Then, the buffer solution surrounding the tissue was collected to quantify the FD-4 that had penetrated the ileal intestinal wall using a 96-well plate reader (BioTek^®^ Synergy HT Microplate reader, BioTek, Santa Clara, CA, USA) at 485 nm excitation and 530 nm emission wavelengths. Data were normalized to the Normal group, which was set as 100%.

### 2.11. Serum Biochemical Marker Measurements

The inflammatory cytokines tumor necrosis factor-alpha (TNF-α), interleukin-1beta (IL-1β), and interleukin-6 (IL-6) were measured using BD OptEIA^™^ Mouse ELISA Kits (BD Biosciences, San Diego, CA, USA) according to the manufacturer’s instructions. Moreover, serum downstream products of inflammation, Prostaglandin E2 (PGE2) and Cyclooxygenase-2 (COX-2), were determined using Assay Genie Mouse ELISA Kits (Assay Genie, Dublin, Ireland) according to the manufacturer’s instructions. Serum total cholesterol (TC), triglyceride (TG), glucose (GLC), blood urea nitrogen (BUN), aspartate aminotransferase (AST), and alanine aminotransferase (ALT) levels were measured by commercial assay kits according to the manufacturer’s instructions (Asan Pharm, Seoul, Republic of Korea).

### 2.12. Gut Microbiota Analysis

After sacrifice, the mice’s cecal feces were collected for analysis of gut microbiota. The QIAGEN DNeasy PowerSoil Kit (Qiagen, Hilden, Germany) was used to isolate bacterial genomic DNA (gDNA) from cecum samples according to the manufacturer’s protocols. The extracted DNA was quantified using a Quant-IT PicoGreen DNA assay kit (Invitrogen, Waltham, MA, USA). A total of 5 ng of gDNA extracted from cecal feces samples was used to construct sequencing libraries following the Illumina 16S Metagenomic Sequencing Library Preparation protocol. The V3-V4 regions of the bacterial 16S rRNA gene were PCR-amplified using Herculase II Fusion DNA Polymerase (Agilent Technologies, Santa Clara, CA, USA), 5× reaction buffer, 1 mM dNTP mix, and 500 nM concentrations of the universal forward/reverse (F/R) PCR primer.

V3-F: 5′-TCG TCG GCA GCG TCA GAT GTG TAT AAG AGA CAG CCT ACG GGN GGC WGC AG-3′.

V4-R: 5′-GTC TCG TGG GCT CGG AGA TGT GTA TAA GAG ACA GGA CTA CHV GGG TAT CTA ATC C-3′.

The first PCR was performed under the following thermal cycling conditions: 3 min at 95 °C for heat activation, followed by 25 cycles of 30 s at 95 °C, 30 s at 55 °C, and 30 s at 72 °C, followed by a 5 min final extension at 72 °C. The PCR products were purified using AMPure XP beads (Agencourt Bioscience, Beverly, MA, USA). Then, 2 μL of the PCR product was used for the second round of PCR by using Nextera XT indexed primers to attach dual indices and Illumina sequencing adapters. This indexing PCR was carried out under the same thermal cycling conditions as the first round, except for 10 amplification cycles. The final PCR products were purified again with AMPure XP beads and quantified using a KAPA Library Quantification Kit (KAPA Biosystems, Wilmington, MA, USA). Finally, the fragment size and quality of the PCR product were assessed using the TapeStation D1000 ScreenTape system (Agilent Technologies, Waldbronn, Germany). The Sequencing was performed by Macrogen (Seoul, Republic of Korea) using the MiSeqTM platform (Illumina, San Diego, CA, USA).

Then, the Illumina MiSeq raw data for each sample were processed through a series of bioinformatic steps. Using Cutadapt (v3.2), the adapters and F/R primer sequences were removed, and the reads were trimmed to 250 bp (forward) and 200 bp (reverse). Next, the DADA2 (v1.18.0) package of the R program (v4.0.3) was used for quality filtering, denoising, merging, and chimera removal. Sequences with an expected error ≥ 2 were excluded, and the remaining reads were denoised by an established error model. After error correction, paired-end reads were merged, and chimeric sequences were removed to generate amplicon sequence variants (ASVs). Finally, ASVs shorter than 350 bp were used for downstream bioinformatic analysis. Each ASV was aligned at ≥99% similarity to the corresponding reference database using the Bayesian classifier (DADA2 v1.18.0), and QIIME2 (version 2023.9) was used for ASV analysis. For alpha-diversity, the ASV count, Shannon, and Gini–Simpson indices were measured. Beta-diversity was evaluated using weighted UniFrac distances, visualized by principal coordinate analysis (PCoA) plots. Statistical significance between the groups was assessed using permutational multivariate analysis of variance (PERMANOVA). In addition, a linear discriminant analysis (LDA) was performed to identify significant differences in the relative abundance of the bacterial composition.

### 2.13. Statistical Analysis

The statistical data were analyzed using GraphPad Prism 8.0 software (GraphPad Software, San Diego, CA, USA). Statistical differences between groups were determined using one-way ANOVA followed by Tukey’s post hoc test. Groups with different lowercase superscript letters (e.g., a, b, c, d) are significantly different (*p* < 0.05), whereas groups sharing the same letter are not significantly different.

## 3. Results

### 3.1. Determination of Vitexin in the PME

Vitexin in the PME was identified on the basis of its chromatographic behavior and UV spectral characteristics (Figure S1). The vitexin standard produced a sharp, well-resolved peak at 37.762 min (Figure S1a), while the PME exhibited a corresponding peak at 37.789 min under the same analytical conditions (Figure S1b). The close agreement in retention time, together with the near-identical DAD-acquired UV spectra, provided definitive confirmation of analyte identity. Using the established calibration model, the vitexin content in the PME was quantified as 1.57 mg/g. The high selectivity of the chromatographic method, combined with the exceptional linearity of the calibration curve, underscores the robustness and analytical reliability of the approach for quantifying vitexin in complex multi-component formulations.

### 3.2. Antioxidative Activity of PME

**Figure 2:**
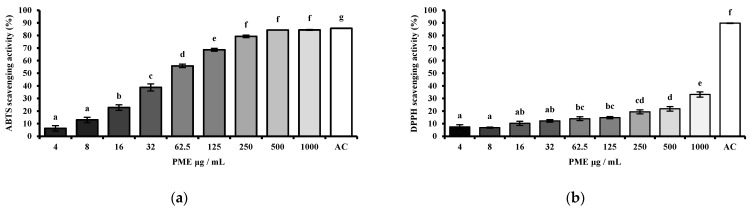
Antioxidant activities of prebiotic mixture extract (PME) at various dosages. (**a**) ABTS scavenging activity; (**b**) DPPH scavenging activity. Results are expressed as mean ± SE (*n* = 4). Different lowercase superscript letters in the same column indicate significant differences (*p* < 0.05).

The antioxidant activity of the PME was determined by measuring the radical-scavenging activities of ABTS and DPPH (Figure 2). ABTS and DPPH are chemical compounds widely used to evaluate the antioxidant activities of plant-derived extracts. The results showed that PME exhibited a concentration-dependent increase in scavenging activity in both the ABTS (Figure 2a) and DPPH (Figure 2b) assays. Although the effect was relatively weak in the DPPH assay, a noticeable increase was observed at 250 μg/mL and higher, with PME showing approximately 30% activity at 1000 μg/mL. Moreover, for ABTS, the scavenging activity increased more steeply with increasing PME concentration than with DPPH. PME reached over 70% scavenging activity at 125 μg/mL and exceeded 80% at concentrations of 250 μg/mL and above. For the positive control, the scavenging activity of ascorbic acid (AC) was greater than 85% in both the DPPH and ABTS assays.

### 3.3. Effect of PME on Proliferation of HT-29 Human Epithelial Cells

**Figure 3:**
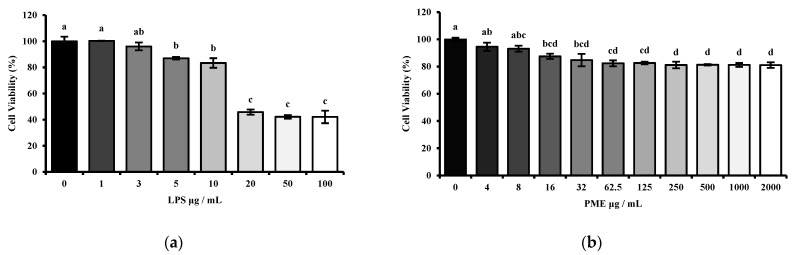
Cell viability rate of HT-29 cells at various dosages of lipopolysaccharide (LPS) and prebiotic mixture extract (PME). (**a**) Cell viability rate of HT-29 cells at various dosages of LPS; (**b**) effect of PME on the cell viability rate of HT-29 cells at various dosages. Results are expressed as mean ± SE (*n* = 4). Different lowercase superscript letters in the same column indicate significant differences (*p* < 0.05).

To determine optimal PME and LPS dosages, MTT assays were performed on HT-29 human epithelial cells. Increasing concentrations of LPS (1, 3, 5, 10, 20, 50, and 100 μg/mL) were used to treat HT-29 cells. As shown in Figure 3a, 10 μg/mL LPS was selected because it significantly induced inflammatory responses without excessive cytotoxicity, thereby providing an appropriate condition for evaluating anti-inflammatory effects.

Moreover, various concentrations (4, 8, 16, 32, 62.5, 125, 250, 500, 750, 1000, 2000 μg/mL) of PME were treated on HT-29 human epithelial cells for 24 h in Figure 3b. PME treatment tended to decrease the viability of the HT-29 cells. However, PME did not significantly affect cell viability at any of the tested concentrations. More than 80% of HT-29 cells remained viable, indicating that the PME is not cytotoxic to intestinal epithelial cells. Therefore, 10 μg/mL of LPS and 100 μg/mL of PME were selected for further co-treatment experiments.

### 3.4. Effect of PME on mRNA Expression Levels of Inflammatory and Tight Junction-Related Markers in LPS-Stimulated HT-29 Human Epithelial Cells

**Figure 4:**
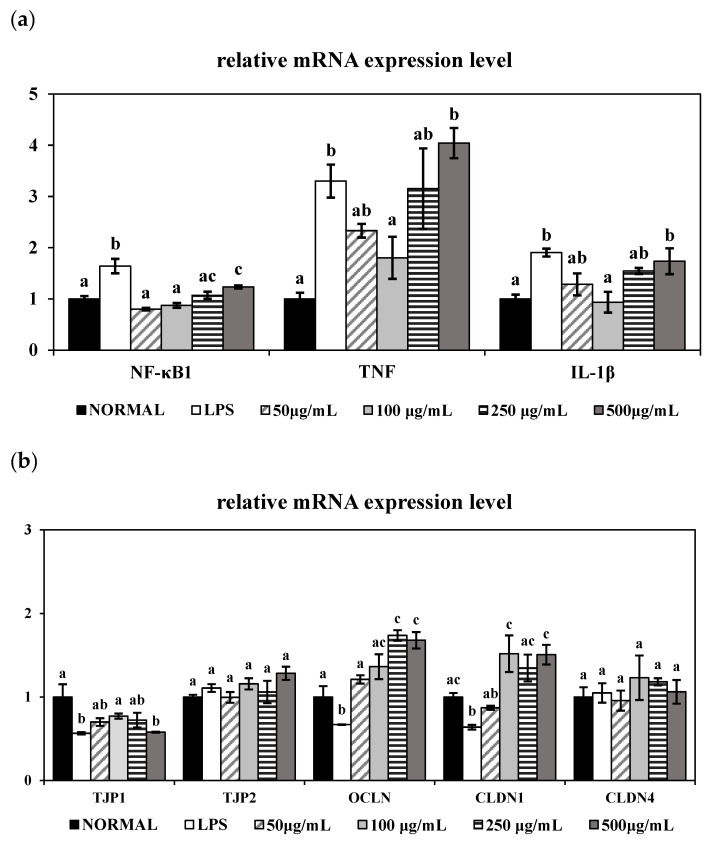
Effect of prebiotic mixture extract (PME) on the mRNA expression levels of inflammatory and tight junction-related markers in LPS-induced HT-29 cells. (**a**) mRNA expression levels of inflammatory markers (nuclear factor-kappa B1 (NF-κB1), tumor necrosis factor-alpha (TNF), and interleukin-1 beta (IL-1β)); (**b**) mRNA expression levels of tight junction-related markers (tight junction protein 1 (TJP1), tight junction protein 2 (TJP2), Occludin (OCLN), Claudin-1 (CLDN1), and Claudin-4 (CLDN4)). Results are expressed as mean ± SE (*n* = 4). Different lowercase superscript letters in the same column indicate significant differences (*p* < 0.05).

The mRNA expression levels of inflammatory markers were observed to confirm the anti-inflammatory property of PME in Figure 4a. Inflammatory markers, TNF-α, IL-1β, and NF-κB1 were evaluated in LPS-stimulated HT-29 human epithelial cells by qRT-PCR. The gene expression levels of inflammatory markers TNF-α and IL-1β, as well as the NF-κB1 transcription factor, were significantly increased (*p* < 0.05) by LPS induction compared to the NORMAL group. Co-treatment with 50 and 100 μg/mL of PME significantly downregulated (*p* < 0.05) the gene expression levels of TNF-α, IL-1β, and NF-κB1. In particular, treatment with 100 μg/mL of PME resulted in a notable reduction in TNF-α, IL-1β, and NF-κB1, with NF-κB1 returning to basal levels. However, treating with over 250 μg/mL of PME increased the gene expression of TNF-α, IL-1β, and NF-κB1, suggesting potential concentration-dependent biphasic effects.

We also examined the gene expression levels of tight junction-related markers TJP1, TJP2, OCLN, CLDN1, and CLDN4 in Figure 4b. LPS stimulation significantly decreased (*p* < 0.05) the gene expression levels of tight junction proteins TJP1, OCLN, and CLDN1 compared to Normal, indicating disrupted epithelial barrier function. TJP1 expression showed a tendency to recover when co-treated with PME at up to 100 μg/mL, but decreased again at higher concentrations (250 and 500 μg/mL), without significance. Interestingly, all PME treatments significantly upregulated OCLN gene expression (*p* < 0.05) compared with the LPS-treated group. Moreover, CLDN1 gene expression was significantly upregulated (*p* < 0.05) by PME treatment, with 100 μg/mL showing the greatest improvement compared to other concentrations. The gene expression levels of TJP2 and CLDN4 did not differ significantly across all groups. Taken together, these findings suggest that PME exerts anti-inflammatory and barrier protective effects in LPS-stimulated HT-29 cells, particularly at 100 μg/mL, by modulating the expression of key inflammatory and tight junction-related genes.

### 3.5. Effect of PME on Growth Performance in DSS-Induced Colitis Mice

The changes in weekly body weight, including percentage differences, are shown in Table 3. The final body weight of DSS-treated mice was significantly lower (*p* < 0.05) than that of the Normal group. Notably, groups administered PME and sulfasalazine (DRUG) tended to regain body weight, but the differences were not statistically significant compared with the DSS group. A similar trend was observed in the percentage change in final body weight; DSS induction significantly decreased the percentage body weight compared to the Normal group (*p* < 0.05). All PME treatments and drugs were capable of restoring the weight rate. However, only the LOW and DRUG groups showed a significant difference (*p* < 0.05) compared to the DSS group. Moreover, the average daily feed intake (ADFI) of all DSS-treated mice significantly decreased (*p* < 0.05). However, no significant differences in average daily water intake (ADWI) were observed between all groups throughout the experimental period.

### 3.6. Effect of PME on Physiological Parameters in DSS-Induced Colitis Mice

**Figure 5:**
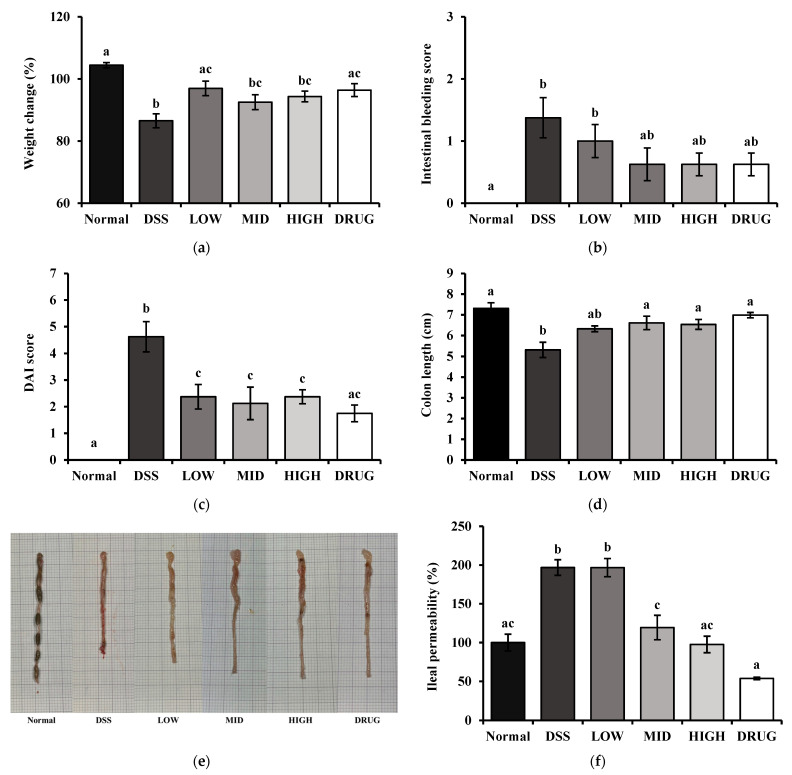
Effect of prebiotic mixture extract (PME) on the physiological parameters in DSS-induced colitis mice. (**a**) Weight change; (**b**) intestinal bleeding score; (**c**) disease activity index (DAI) score; (**d**) colon length; (**e**) colon tissue morphology; and (**f**) ileal permeability. Results are expressed as mean ± SE (*n* = 8). Different lowercase superscript letters in the same column indicate significant differences (*p* < 0.05).

The effects of PME on physiological parameters related to colitis are shown in Figure 5. The changes in weight, disease activity index (DAI) score, and colon length were measured. The weight change percentage was significantly decreased (*p* < 0.05) in the DSS group compared to the Normal group (Figure 5a). In contrast, all PME-treated groups and the DRUG group exhibited partial recovery in body weight; however, only the LOW and DRUG groups showed a significant increase (*p* < 0.05) in weight change percentage compared to the DSS group.

Moreover, the score for intestinal bleeding was significantly higher (*p* < 0.05) in the DSS group compared to the Normal group. In contrast, the PME and DRUG groups showed a tendency toward decreased bleeding scores but did not differ significantly from either the Normal or DSS groups (Figure 5b).

Furthermore, we calculated the DAI score by summing the weight-loss and intestinal-bleeding scores at the end of the experimental period (Figure 5c). The DAI score in the DSS group was significantly higher (*p* < 0.05) than in all groups. Interestingly, all the PME-treated groups and the DRUG group showed significantly lower DAI scores (*p* < 0.05) than the DSS group, with the DRUG group displaying levels statistically comparable to the Normal group.

Colon length, which is a representative indicator of colonic inflammation and tissue damage, was significantly shortened (*p* < 0.05) in the DSS group compared to the Normal group (Figure 5d,e). PME treatment attenuated this reduction in a dose-dependent manner, with the MID and HIGH groups showing significantly longer (*p* < 0.05) colons than the DSS group. The DRUG group also recovered colon length to a level similar to that of the Normal group, indicating the protective effect of PME against DSS-induced colonic shortening.

Ileal permeability, which is an indicator of intestinal barrier integrity, is shown in Figure 5f. DSS treatment significantly increased (*p* < 0.05) permeability compared with the Normal group, reflecting impaired gut barrier function. The LOW group showed no significant improvement from DSS, maintaining high permeability levels. On the other hand, the MID and HIGH groups significantly reduced permeability rates compared to the DSS group (*p* < 0.05). Interestingly, the DRUG group exhibited the lowest permeability among all groups, similar to the Normal group, suggesting an intestinal protective effect. Taken together, these results indicate that PME may exert protective effects against DSS-induced colitis by restoring physiological and intestinal barrier functions.

### 3.7. Effect of PME on Histological Parameters in DSS-Induced Colitis Mice

**Figure 6:**
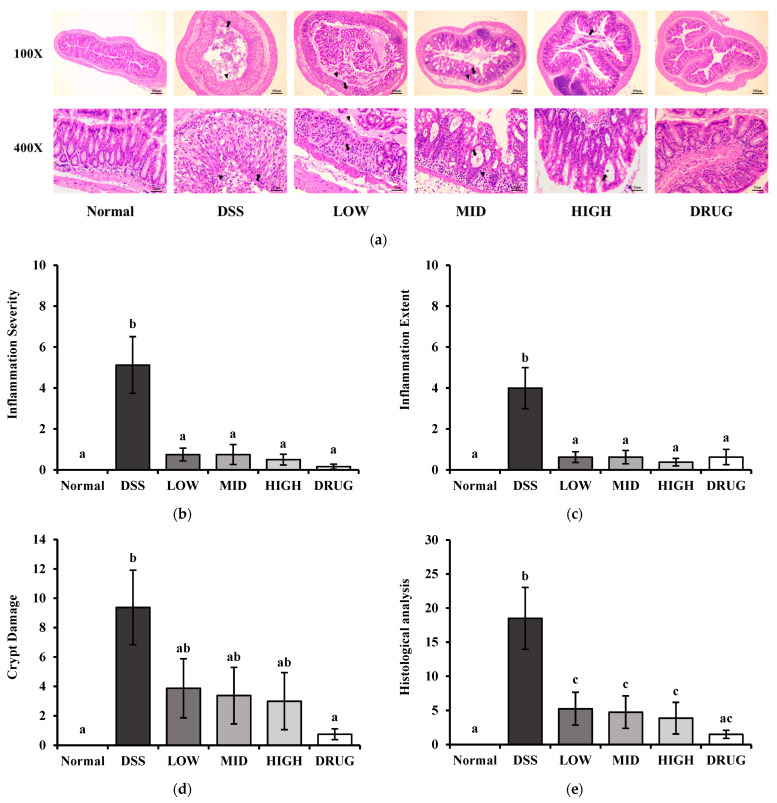
Effect of prebiotic mixture extract (PME) in DSS-induced colitis mice assessed by H&E staining. (**a**) H&E-stained distal colon section (100× top and 400× bottom). Arrow and arrowheads indicate crypt damage, erosion of the epithelium, and cell infiltration; (**b**) inflammation severity score; (**c**) inflammation extent score; (**d**) crypt damage score; and (**e**) histological analysis score. Results are expressed as mean ± SE (*n* = 8). Different lowercase superscript letters in the same column indicate significant differences (*p* < 0.05).

To further confirm the ameliorating effect of PME, colonic tissue-level damage was assessed by hematoxylin and eosin (H&E) staining (Figure 6a). The DSS group showed severe colonic disruption characterized by crypt disappearance, inflammatory infiltration, and erosion in epithelial surfaces. As shown in Figure 6b,c, increased intestinal inflammation scores induced by DSS were significantly decreased (*p* < 0.05) by PME and DRUG treatments. Moreover, the increased crypt damage rate stimulated by DSS was also alleviated by PME and DRUG; however, only DRUG showed significance compared to DSS (*p* < 0.05) (Figure 6d). Additionally, histological analysis scores of PME and DRUG groups were significantly lower than those of DSS groups (Figure 6e). Detailed numerical values for histopathological scoring, including epithelial damage and inflammatory infiltration scores, are provided in Table S1. Taken together, these results indicate that PME can prevent intestinal inflammation and crypt damage in the DSS-induced colitis mouse model.

### 3.8. Effect of PME on Serum Biochemical Markers in DSS-Induced Colitis Mice

**Figure 7:**
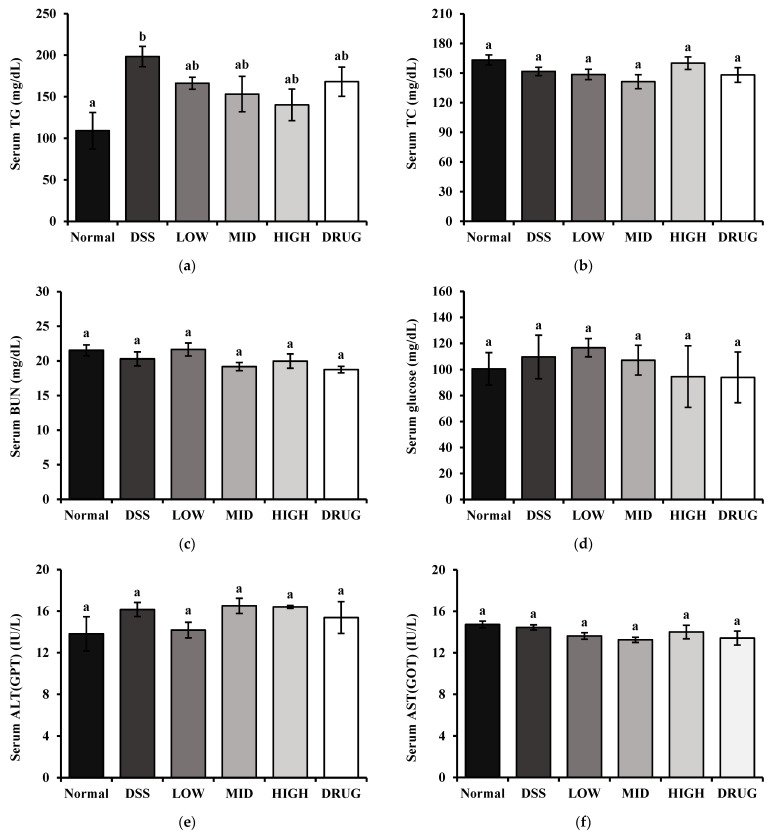
Effect of prebiotic mixture extract (PME) on the serum biochemical markers related to body metabolism in DSS-induced colitis mice. (**a**) Triglyceride (TG); (**b**) total cholesterol (TC); (**c**) blood urea nitrogen (BUN); (**d**) glucose; (**e**) alanine transaminase (ALT/GPT); and (**f**) aspartate transaminase (AST/GOT). Results are expressed as mean ± SE (*n* = 8). Different lowercase superscript letters in the same column indicate significant differences (*p* < 0.05).

The effects of PME on serum biochemical markers related to metabolic activity are illustrated in Figure 7. As shown in Figure 7a, serum triglyceride (TG) levels were significantly increased (*p* < 0.05) by DSS induction. Interestingly, PME treatment tended to reduce serum TG levels in a dose-dependent manner; however, the differences were not statistically significant. No significant differences were observed among the groups in serum total cholesterol (TC), blood urea nitrogen (BUN), glucose, alanine aminotransferase (ALT), and aspartate aminotransferase (AST), suggesting that PME had no significant effect on serum biochemical markers, indicating that it did not induce metabolic dysfunction (Figure 7b–f).

**Figure 8:**
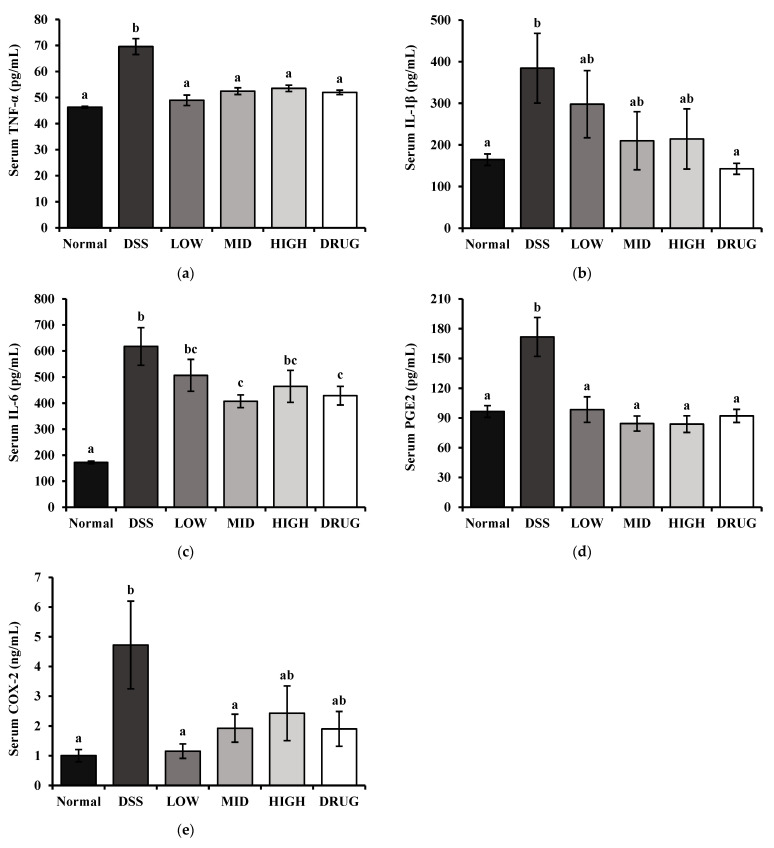
Effect of prebiotic mixture extract (PME) on the serum biochemical markers related to inflammation in DSS-induced colitis mice. (**a**) Tumor necrosis factor-alpha (TNF-α); (**b**) interleukin-1beta (IL-1β); (**c**) interleukin-6 (IL-6); (**d**). Prostaglandin E2 (PGE_2_) and (**e**) Cyclooxygenase 2 (COX-2). Results are expressed as mean ± SE (*n* = 8). Different lowercase superscript letters in the same column indicate significant differences (*p* < 0.05).

Furthermore, the effect of PME on serum biochemical markers related to inflammatory activity is shown in Figure 8. Serum levels of TNF-α, IL-1β, IL-6, PGE_2_, and COX-2 were measured to determine the effects of PME on inflammatory activities in DSS-induced mice. The serum levels of the pro-inflammatory cytokines TNF-α, IL-1β, and IL-6 were significantly increased (*p* < 0.05) in the DSS group compared to the Normal group (Figure 8a–c). PME treatment at all doses showed a trend toward reducing pro-inflammatory cytokine levels. Serum TNF-α was significantly decreased (*p* < 0.05) in all PME-treated groups and the DRUG group. However, a significant reduction in serum IL-1β (*p* < 0.05) was observed only in the DRUG group. Additionally, serum IL-6 levels were significantly lower in the MID and DRUG groups than in the DSS group. To further assess the inflammatory response, serum levels of Prostaglandin E_2_ (PGE_2_) and Cyclooxygenase-2 (COX-2) were measured (Figure 8d,e). Serum PGE_2_ levels were significantly elevated (*p* < 0.05) in the DSS group compared with the Normal group, whereas all PME-treated and DRUG groups maintained significantly lower levels, comparable to those of the Normal group. Similarly, serum COX-2 levels were significantly increased (*p* < 0.05) in the DSS group. PME treatment at LOW and MID doses significantly suppressed (*p* < 0.05) COX-2 expression compared to the DSS group, while the HIGH and DRUG groups showed a partial, but not statistically significant, reduction. This suggests that PME may exert a downregulatory effect on DSS-induced inflammation.

### 3.9. Effect of PME on the Alteration of Gut Microbiota Composition in DSS-Induced Colitis Mice

**Figure 9:**
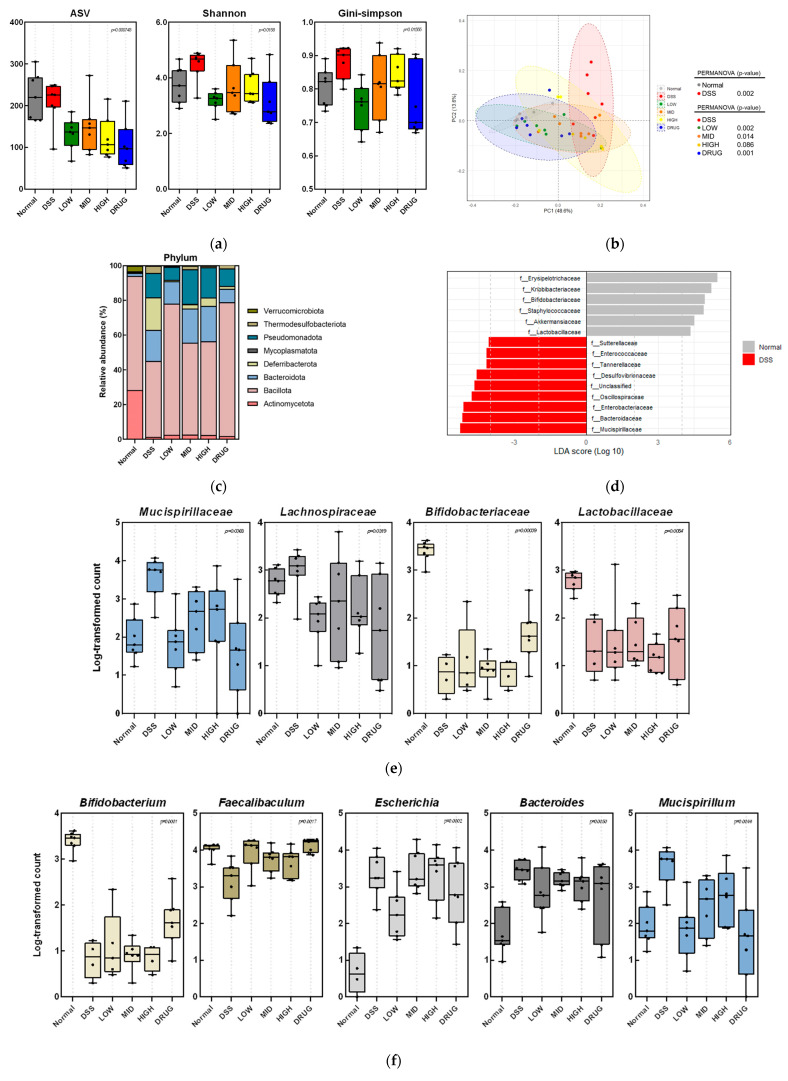
Effect of prebiotic mixture extract (PME) on altered gut microbiota composition in DSS-induced colitis mice. (**a**) Alpha−diversity; (**b**) two−dimensional PCoA plot of beta-diversity based on the weighted UniFrac distance; (**c**) relative abundance of bacterial composition at the phylum level; (**d**) LDA scores at the family level; (**e**) relative log−transformed count at the family level; and (**f**) relative log−transformed count at the genus level.

The correlation between the intestinal attenuating effect of PME and the gut microbiota was determined by sequencing the 16S rRNA gene from fecal samples collected from DSS-induced colitis mice (Figure 9). To investigate alpha-diversity of the intestinal microbiota, ASV (amplicon sequence variant), Shannon, and Gini–Simpson indices were calculated. As shown in Figure 9a, alpha-diversity indices, including Shannon and Simpson, were increased in the DSS group compared to the Normal group. However, PME treatments and DRUG showed decreasing trends in ASV, Shannon, and Gini-Simpson indices. Then, the beta-diversity of the gut microbiota, based on weighted UniFrac distances, was visualized by principal coordinates analysis (PCoA) and revealed clear separation between the Normal and DSS groups (Figure 9b). Interestingly, the clustering pattern of the PME- and DRUG-treated groups approached that of the Normal group, whereas the LOW and DRUG groups were completely separated from the DSS group.

Moreover, alterations in gut microbiota composition at various taxonomic levels are shown in Figure 9c–f. At the phylum level, DSS stimulation decreased Bacillota (formerly Firmicutes) and increased the abundances of Bacteroidota (formerly Bacteroidetes) and Pseudomonadota (formerly Proteobacteria) compared to the Normal group (Figure 9c). However, the PME and DRUG groups showed a recovery trend in Bacillota levels, which closely resembled that of the Normal group.

Next, a linear discriminant analysis (LDA) was performed to confirm gut microbial alterations at the family level, revealing a significant difference with a log LDA score of 4.0 (Figure 9d). In particular, increased *Mucispirillaceae* and *Lachnospiraceae* in the DSS group were partially reversed, approaching levels similar to those of the Normal group in PME and DRUG groups (Figure 9e). Decreased *Bifidobacteriaceae* and *Lactobacillaceae* in the DSS group showed no significant difference with PME treatment. However, *Bifidobacteriaceae* in the DRUG group showed a gradual reversal toward the Normal group.

At the genus level, *Bifidobacterium* and *Faecalibaculum* were significantly reduced in the DSS group (Figure 9f). Only *Bifidobacterium* was recovered in the DRUG group, whereas *Faecalibaculum* was recovered to levels similar to the Normal group in all PME and DRUG-treated groups. *Escherichia* was increased in the DSS group compared to the Normal group, but only a low dose of PME was capable of reversing the increased level of *Escherichia* in the Normal group. *Bacteroides* was also increased due to DSS. Interestingly, the increased level of *Bacteroides* by DSS was restored by PME treatments in a dose-dependent manner. Moreover, *Mucispirillum* was also elevated by DSS, while treatment with a low dose of PME and DRUG gradually reduced its abundance.

## 4. Discussion

In this study, a hot-water extract mixture of *Vigna radiata* (mung bean), *Vigna angularis* (red bean), and *Foeniculum vulgare* (fennel) has been shown to improve gastrointestinal barrier function in both in vitro and in vivo models. Previous studies have shown the intestinal protective effects of *Vigna radiata* (mung bean), *Vigna angularis* (red bean), and *Foeniculum vulgare* (fennel) [11,12,13,14]. Therefore, we expected that the combined extract of *Vigna radiata* (mung bean), *Vigna angularis* (red bean), and *Foeniculum vulgare* (fennel) would exhibit a synergistic effect on intestinal health.

To evaluate the antioxidant activity of PME, DPPH and ABTS assays were used, which are widely used methods for measuring the scavenging activities of plant-derived extracts. In our study, PME showed dose-dependent radical-scavenging effects in both assays, with ABTS activity higher than DPPH. At concentrations above 125 μg/mL, PME reached over 70% scavenging in the ABTS assay, while DPPH activity remained below 30% even at 1000 μg/mL. Since PME was extracted with hot water, it is likely to contain hydrophilic antioxidant compounds that are more reactive with ABTS radicals. This aligns with previous findings that the ABTS radical is soluble in both aqueous and organic solvents and can interact with both hydrophilic and hydrophobic antioxidants, whereas the DPPH radical primarily reacts with hydrophobic antioxidants [24]. These data indicate that PME exhibited moderate antioxidant potential, showing its high efficiency in scavenging free radicals.

Then, the appropriate concentrations of LPS and PME were determined by cytotoxicity assessment using MTT assays in HT-29 human intestinal epithelial cells. The LPS dosage range was selected between 1 μg/mL and 100 μg/mL based on previous studies [25,26,27,28]. Our results showed that LPS treatment at 10 μg/mL significantly reduced cell viability compared to the untreated control, while keeping cell viability above 80%. This concentration was therefore considered optimal, as it was sufficient to induce an inflammatory response without causing excessive cytotoxicity in epithelial cells.

Moreover, PME exhibited decreased cell viability in a dose-dependent manner. More than 80% of cells remained viable even at the highest concentration tested, indicating PME’s low cytotoxicity. Several studies have reported cytotoxic effects of herbal hot water extracts in HT-29 and other cancer cell models. For example, water extracts of *Manilkara zapota* and *Drimia calcarata* were reported to possess cytotoxicity towards HT-29 cells [29,30]. These findings highlight the importance of conducting cytotoxicity screening before using herbal extracts in therapeutic or mechanistic studies. Therefore, 10 μg/mL of LPS and 100 μg/mL of PME were selected as working concentrations for further cell experiments based on these findings.

It is well established that LPS triggers TNF-α and IL-1β release and NF-κB activation in colonic epithelial cells, thereby inducing intestinal epithelial damage [31]. Consistent with previous findings, LPS significantly upregulated the mRNA expression levels of TNF-α, IL-1β, and NF-κB in HT-29 cells. However, co-treatment with 100 μg/mL of PME effectively suppressed the expression of the inflammatory markers TNF-α and IL-1β and downregulated the transcription factor NF-κB1, indicating the anti-inflammatory property of PME. Interestingly, treatment with higher concentrations of PME (250 and 500 μg/mL) led to a re-elevation of TNF-α, IL-1β, and NF-κB gene expression, suggesting a dose-dependent biphasic effect of PME. A study by Barreiro-Sisto et al. demonstrated that polyphenolic and plant-derived compounds exhibit hormetic dose–response curves, in which low or moderate doses confer anti-inflammatory benefits. In contrast, higher doses paradoxically exacerbate inflammation or induce cellular stress, leading to the activation of pro-inflammatory pathways, such as NF-κB, and increased cytokine production [32]. In the case of PME, low to moderate concentrations may activate anti-inflammatory pathways and confer beneficial effects, whereas higher concentrations may trigger cellular stress, amplifying inflammatory responses. This concentration-dependent effect underscores the importance of optimizing the dosage when using plant-derived extracts, as excessive amounts may undermine their beneficial properties and exacerbate inflammation.

To further assess the protective effects of PME on intestinal epithelial integrity, we examined the gene expression levels of key tight junction markers in LPS-stimulated HT-29 cells. Our study showed effective restoration of OCLN and CLDN1 gene expression in LPS-treated HT-29 cells, consistent with previous in vivo studies using *Vigna radiata* (mung bean), *Vigna angularis* (red bean), and *Foeniculum vulgare* (fennel). Ao et al. demonstrated that bound polyphenols derived from *Vigna radiata* (mung bean)-coated dietary fiber ameliorated DSS-induced ulcerative colitis in mice by increasing intestinal barrier function via upregulation of tight junction marker gene expression, including OCLN and ZO-1 [12]. Moreover, *Foeniculum vulgare* (fennel) seed extracts have also been shown to improve epithelial barrier integrity and upregulate OCLN and ZO-1 in an inflammation-induced cell model by alleviating NF-κB signaling activation [14]. Lastly, *Vigna angularis* (red bean) seed coat extracts have shown their protective effects in colitis models. For example, red bean extracts alleviated DSS-induced acute colitis by reducing inflammation and restoring OCLN expression [13]. To our knowledge, while in vivo studies have demonstrated that *Vigna radiata* (mung bean), *Vigna angularis* (red bean), and *Foeniculum vulgare* (fennel) can restore tight junction proteins in murine colitis models, no cellular experiments using HT-29 or similar epithelial cells have yet been reported. Therefore, our present findings provide novel in vitro evidence supporting the intestinal barrier protective activity of the combined PME.

Taken together, our results align with previous in vivo findings showing that combined PME can restore the inflammatory response and tight junctions. In particular, treatment with 100 μg/mL of PME effectively reduced TNF-α and IL-1β expression, while concurrently enhancing CLDN1 and OCLN expression in LPS-induced human epithelial cells. This finding suggests the combined extract of *Vigna radiata* (mung bean), *Vigna angularis* (red bean), and *Foeniculum vulgare* (fennel) may confer a synergistic advantage when administered at an optimal concentration. Therefore, to further validate these in vitro observations, we next evaluated the protective effects of PME in a DSS-induced colitis mouse model at various dosage levels.

The DSS-induced mouse model closely mimics the pathophysiological features of human ulcerative colitis, including mucosal inflammation, epithelial damage, and weight loss [33]. Therefore, the DSS-induced mouse colitis model is widely used before human trials of IBD treatments. In this study, we evaluated the intestinal health-promoting effect of PME in the colitis mouse model. PME was capable of ameliorating DSS-induced colitis symptoms by normalizing serum biomarkers of inflammation and altering gut microbiota composition. Moreover, PME restored impaired intestinal phenotype parameters, including colon length, ileal permeability, and DAI scores.

DSS-induced colitis typically results in significant weight loss in mice, a reliable and objective indicator of disease severity. This weight loss is attributed to a combination of impaired nutrient absorption and malnutrition associated with colonic inflammation [34]. In the present study, DSS-treated mice exhibited a significant decrease in final body weight and percentage weight change compared with the Normal group, consistent with previous findings [11,12,35]. Although PME-treated groups and the sulfasalazine-administered DRUG group showed a tendency to recover body weight, only the LOW and DRUG groups exhibited statistically significant improvements in weight change percentage. It has been reported that this partial restoration may be associated with the alleviation of colonic inflammation and enhancement of nutrient utilization [36]. Additionally, average daily feed intake (ADFI) was significantly reduced in all DSS-treated mice, consistent with prior reports indicating that DSS reduces appetite and induces discomfort during the colitis phase [37]. However, average daily water intake (ADWI) remained unchanged across all groups, suggesting that DSS-induced systemic illness primarily affects appetite, thereby leading to decreased body weight. Moreover, to evaluate potential systemic or metabolic adverse effects of PME, we measured serum biomarkers of body metabolism, including glucose, TG, TC, BUN, AST, and ALT, in DSS-induced mice. All DSS- and PME-treated groups showed no significant differences in serum metabolic biomarker levels, except for TG. Interestingly, serum TG levels were significantly increased by DSS induction, and PME tended to normalize these elevated TG levels in a dose-dependent manner. A previous report demonstrated that hepatic TG levels were significantly increased in DSS-induced colitis mice [38]. These results suggest that the observed TG elevation may reflect a DSS-associated metabolic disruption rather than a direct effect of PME. Overall, our analysis revealed no significant changes in body metabolism-related indicators, suggesting that PME does not compromise metabolic homeostasis. These findings thereby support the safety profile of PME as a therapeutic candidate for colitis.

Colitis induced by DSS is characterized by marked weight loss, intestinal bleeding, colonic shortening, and increased intestinal permeability, collectively reflecting the severity of mucosal inflammation and disruption of gut barrier integrity [38,39]. In the present study, mice exposed to DSS exhibited significant reductions in body weight and increased intestinal bleeding, leading to elevated disease activity index (DAI) scores, consistent with previously reported colitis models [37,38]. However, in our study, administration of PME reversed these symptoms by showing significant improvements in body weight maintenance and exhibiting significantly lower DAI scores compared to the negative control group (DSS-only-treated group). It is well known that colon length and ileal permeability rate reflect mucosal damage and barrier dysfunction [40]. Our study also demonstrated that colon length was significantly preserved in mid- and high-dose PME-treated mice, comparable to that observed in the DRUG group. These structural improvements were accompanied by restoration of intestinal barrier function, as evidenced by significantly reduced ileal permeability in the MID, HIGH, and DRUG groups. Elevated ileal permeability in the DSS group reflects impaired tight junctions in the gut, indicating gut barrier dysfunction. The ability of PME to suppress ileal permeability indicates its role in maintaining mucosal integrity, possibly by modulating tight junction protein expression or inflammatory pathways.

To further examine the intestinal health-promoting effect of PME, inflammation scores and crypt damage in the colonic mucosa were assessed by H&E staining. Colitis induced by DSS leads to severe intestinal inflammation, characterized by infiltration of immune cells and tissue swelling. It causes damage to the crypt structures within the colonic mucosa, thereby impairing normal epithelial organization and function [22]. Histological examination of colon sections stained with H&E revealed that treatment with either DRUG or PME alleviated the pathological features of DSS-induced colitis, including excessive inflammatory cell infiltration and structural injury to the mucosal crypts. Interestingly, mice treated with a high dosage of PME exhibited the most pronounced protective effect, showing the lowest rate of inflammatory cell infiltration among the experimental groups.

Inflammatory cytokines, such as TNF-α, IL-1β, and IL-6, are central mediators of the mucosal immune response and are closely linked to the pathogenesis of colitis-stimulated inflammation [11]. Therefore, we measured these inflammatory factors in serum by ELISA to evaluate whether PME treatment modulates the systemic inflammatory response in DSS-induced colitis. Our study showed that PME treatment effectively suppressed several of these inflammatory markers. In particular, serum TNF-α levels were significantly reduced across all PME doses, highlighting PME’s anti-inflammatory potential. Meanwhile, PME administration tended to lower serum IL-1β and IL-6 levels; however, the reductions did not reach statistical significance, except for serum IL-6 in the MID group. In addition, PGE_2_ and its upstream regulator, COX-2, are key inflammatory mediators that contribute further to epithelial damage and intestinal barrier dysfunction [41]. Moreover, COX-2 deficiency itself has been reported to exacerbate intestinal epithelial permeability and promote bacterial translocation, further underscoring the protective role of COX-2 in gut barrier maintenance [42]. In our study, serum levels of both PGE_2_ and COX-2 were significantly decreased by PME treatment, especially in the LOW and MID groups. This data is consistent with our ileal permeability result, further supporting the connection between COX-2 signaling and impaired epithelial barrier function. These results suggest that PME ameliorates DSS-induced colitis not only by suppressing classical pro-inflammatory cytokines but also by modulating the COX-2/PGE_2_ pathway.

Vitexin is a naturally occurring C-glycosylated flavone that has been shown to exert significant antioxidant and anti-inflammatory properties [18]. Specifically, vitexin has been reported to reduce oxidative stress by scavenging free radicals, suppress the production of pro-inflammatory cytokines like TNF-α and IL-6, and modulate key inflammatory pathways, including COX-2/PGE_2_ [19]. Therefore, vitexin was selected for analysis because of its well-established role as a major bioactive compound in legumes and medicinal plants, as well as its significant effects on oxidative stress and inflammation [18]. In our study, vitexin was quantified at 1.57 mg/g in the PME, suggesting a significant contribution to the observed gut-protective effects. However, it is important to explore whether other bioactive compounds are also present in the PME, as they may contribute to its overall efficacy. Future studies should focus on identifying and quantifying these additional bioactive compounds to clarify the synergistic effects of the PME. Understanding the full profile of bioactive compounds in PME will provide deeper insights into its mechanism of action and enhance its therapeutic potential.

Finally, we investigated the diversity of gut microbiota composition in the PME-treated mouse colitis model. In our study, DSS-induced colitis increased alpha-diversity indices, which is inconsistent with the general trend of reduced microbial diversity reported in IBD patients [43,44]. We speculate that this paradoxical increase may be associated with the disruption of intestinal barrier integrity caused by DSS treatment. Damage to the epithelial and mucus layers can enhance intestinal permeability, facilitating the translocation of luminal bacteria and their metabolites into submucosal tissues [45]. Previous studies have demonstrated that DSS-induced intestinal injury compromises the colonic mucus and epithelial barriers, thereby promoting bacterial translocation and even bacteremia in mice [46]. Such perturbations of the mucus and epithelial layers may substantially alter the gut microenvironment, allowing the expansion of opportunistic or previously suppressed microbial taxa [47]. Consequently, this dysregulated microbial overgrowth could manifest as an apparent increase in species richness (alpha-diversity), reflecting barrier dysfunction rather than improved microbial health. Therefore, detailed taxonomic profiling at the phylum, family, and genus levels was needed to better understand how our results differ from those of earlier studies. Next, beta-diversity analysis also revealed a distinct separation between the DSS and Normal groups, confirming that DSS-induced colitis caused a marked alteration in the overall microbial community structure. Interestingly, PME and DRUG treatments altered the microbial community toward that of the Normal group, suggesting partial recovery of the intestinal microbiota. Notably, the LOW and DRUG groups showed the greatest separation from the DSS group, suggesting a strong modulatory effect on microbial structure. This may reflect a transient expansion of opportunistic taxa under inflammatory stress rather than genuine microbial stability [48]. PME and DRUG treatments reduced these indices toward normal levels, suggesting the restoration of a more balanced and stable microbial community structure.

To investigate detailed microbial alterations, the gut microbiota composition was further examined at the phylum, family, and genus levels. In our study, DSS treatment decreased the abundance of Bacillota (formerly Firmicutes). It increased the abundance of Bacteroidota and Pseudomonadota (formerly Bacteroidetes and Proteobacteria), which are consistent with dysbiotic patterns observed in both DSS-induced colitis models and IBD patients [49]. However, LOW doses of PME and DRUG treatments exhibited a restorative trend in Bacillota abundance, approaching the levels observed in the Normal group. This recovery may indicate the re-establishment of butyrate-producing and barrier-protective bacteria, which are essential for maintaining intestinal homeostasis. Meanwhile, the relative abundance of Pseudomonadota decreased following low doses of PME and DRUG administration, suggesting suppression of inflammation-associated taxa [50].

At the family level, the abundance of *Mucispirillaceae* and *Lachnospiraceae* was markedly elevated in the DSS group. In contrast, LOW doses of PME and DRUG treatments normalized this family’s abundance, indicating recovery toward a balanced microbial community. *Mucispirillaceae* is known to be associated with mucin degradation and inflammatory conditions [51], and increased abundance of *Lachnospiraceae* under inflammatory stress has been reported as resulting in microbial imbalance [52]. Moreover, beneficial families *Bifidobacteriaceae* and *Lactobacillaceae* were significantly depleted by DSS administration, as observed in previous studies [53,54]. Low doses of PME and DRUG treatments partially restored their levels, implying the re-establishment of beneficial bacterial populations that contribute to butyrate production and epithelial barrier maintenance [55].

Lastly, at the genus level, *Bifidobacterium* and *Faecalibaculum* were significantly decreased in the DSS group, a pattern frequently observed in IBD patients and colitis models [56,57]. Treatment with a low dose of PME and DRUG partially restored *Faecalibaculum* to near normal levels, whereas a significant recovery of *Bifidobacterium* was observed mainly in the DRUG group. *Bifidobacterium* is a well-known beneficial bacterium; however, *Faecalibaculum* primarily produces lactate and acetate, which may serve as substrates for butyrate-producing bacteria, thereby indirectly contributing to butyrate production and epithelial protection [58]. Conversely, the abundances of *Escherichia* and *Bacteroides* were increased in the DSS group, indicating the expansion of inflammation-associated bacteria belonging to the Pseudomonadota and Bacteroidota phyla. Previous studies have reported that *Escherichia* and *Bacteroides* are enriched in oxidative and inflammatory environments, and their expansion represents a typical dysbiotic signature in both murine colitis models and IBD patients [59]. Notably, PME treatments significantly reduced Bacteroides abundance in a dose-dependent manner. At the same time, only a low dose of PME was effective in suppressing *Escherichia*, suggesting a selective regulatory effect. Finally, *Mucispirillum*, a genus in the *Mucispirillaceae* family, was also elevated by DSS stimulation, a genus known for mucin degradation [51]. Our study showed that LOW doses of PME and DRUG treatments attenuated this abnormal increase, implying the recovery of mucosal integrity.

Although mid and high doses of PME improved host parameters such as colon length, ileal permeability, and serum inflammatory markers more effectively, we found that the low dose produced a more favorable gut microbiota, characterized by increased *Bifidobacterium* and *Faecalibaculum*, along with reduced *Escherichia* and *Mucispirillum*. This discrepancy between microbial and physiological results may indicate a nonlinear, dose-dependent response, where low-dose PME primarily exerts prebiotic effects by selectively enriching beneficial taxa and restoring microbial balance. In contrast, higher doses may act more directly on host tissues, reducing inflammation and enhancing barrier integrity through molecular mechanisms that are partially independent of gut microbiota. The discrepancy may also reflect a temporal gap between microbiota modulation and histological improvement, resulting in distinct outcomes depending on the treatment dose. Similarly, to our study, Mysonhimer and colleagues reported that prebiotic supplementation with fructooligosaccharides and galactooligosaccharides significantly altered gut microbiota composition in healthy adults; however, they observed no measurable changes in inflammatory or stress-related biomarkers, suggesting a discrepancy between microbial alterations and host physiological outcomes [60]. Moreover, Ferenczi and colleagues demonstrated that oligomannan supplementation in colitic mice alleviated immunological and inflammatory markers; however, it did not fully normalize histological parameters, despite alterations in gut microbiota [61]. These findings support our finding that microbial restructuring can occur independently of systemic physiological improvements, as observed in the LOW dose group. Collectively, our data suggest a dose-dependent dual action of PME, where low-dose treatment primarily influences gut microbial composition, and higher doses contribute to inflammation reduction and epithelial protection; however, further studies are needed to clarify the mechanisms underlying these dose-dependent effects.

## 5. Conclusions

In conclusion, the present study demonstrated that the hot-water extract mixture of *Vigna radiata* (mung bean), *Vigna angularis* (red bean), and *Foeniculum vulgare* (fennel) exerts potential protective effects against intestinal inflammation and barrier dysfunction, both in vitro and in vivo. PME exhibited antioxidant and anti-inflammatory properties by suppressing LPS-induced TNF-α, IL-1β, and NF-κB expression, while enhancing tight junction-related genes such as *CLDN1* and *OCLN* in HT-29 epithelial cells. Furthermore, PME ameliorated DSS-induced colitis in mice by improving clinical parameters, including body weight, colon length, and intestinal permeability, and by reducing serum levels of inflammatory cytokines and COX-2/PGE2. In addition, the anti-inflammatory effects of PME are likely mediated by vitexin, which has been shown to reduce oxidative stress and modulate key inflammatory pathways such as COX-2/PGE_2_. Notably, PME restored the composition of gut microbiota disrupted by DSS treatment, promoting beneficial taxa (*Bifidobacterium*, *Faecalibaculum*) while suppressing inflammation-associated bacteria (*Escherichia*, *Bacteroides*, *Mucispirillum*). These findings suggest that PME enhances intestinal barrier integrity and modulates gut microbial homeostasis, providing scientific evidence that the synergistic combination of mung bean, red bean, and fennel may serve as a promising dietary or therapeutic approach for managing colitis and maintaining gut health. Therefore, our findings suggest that the low and middle doses of PME are the most effective concentrations for exerting a gut-protective effect, and ongoing in vivo studies aim to clarify its molecular mechanisms.

## Figures and Tables

**Table 1:** General information on Vigna radiata, Vigna angularis, and Foeniculum vulgare.

Common Name	Scientific Name	Key Characteristics	Use/Applications
Mung bean	*Vigna radiata*	High in protein and vitamins B and C	Used in traditional medicine for digestive health
Red bean	*Vigna angularis*	Rich in antioxidants and protein	Used in traditional medicine for diabetes management
Fennel	*Foeniculum vulgare*	Rich in fiber and vitamin C	Used for digestive disorders and anti-inflammatory effects

**Table 2:** Gene-target and TaqMan primer assays in LPS-induced HT-29 human epithelial cells.

Gene Name	TaqMan Probes	Assay ID
TNF	Tumor necrosis factor α	Hs00174128_m1
IL-1β	Interleukin 1 beta	Hs01555410_m1
NF-κB1	Nuclear factor kappa B subunit 1	Hs00765730_m1
TJP1	Tight junction protein 1 (Zonula occludens-1)	Hs01551871_m1
TJP2	Tight junction protein 2 (Zonula occludens-2)	Hs00910543_m1
OCLN	Occludin	Hs05465837_g1
CLDN1	Claudin-1	Hs00221623_m1
CLDN4	Claudin-4	Hs00976831_s1
GAPDH	Glyceraldehyde-3-phosphate dehydrogenase	Hs03929097_g1

**Table 3:** Growth performance of DSS-induced colitis mice.

	Normal	DSS	LOW	MID	HIGH	DRUG
Initial body weight (g) Day 0	26.55 ± 0.41 ^a^	26.23 ± 0.22 ^a^	26.53 ± 0.57 ^a^	26.25 ± 0.36 ^a^	26.26 ± 0.31 ^a^	26.37 ± 0.33 ^a^
DSS initiation body weight (g) Day 7	30.21 ± 0.62 ^a^	30.07 ± 0.67 ^a^	30.44 ± 0.72 ^a^	30.26 ± 0.72 ^a^	29.86 ± 0.54 ^a^	29.27 ± 0.66 ^a^
Final body weight (g) Day 14	31.56 ± 0.76 ^a^	26.49 ± 1.19 ^b^	29.48 ± 0.86 ^ab^	28.02 ± 1.09 ^ab^	28.16 ± 0.67 ^ab^	28.23 ± 1.01 ^ab^
Initial body weight (%) Day 0	100 ± 0 ^a^	100 ± 0 ^a^	100 ± 0 ^a^	100 ± 0 ^a^	100 ± 0 ^a^	100 ± 0 ^a^
DSS initiation body weight (%) Day 7	112.8 ± 1.2 ^a^	114.5 ± 1.9 ^a^	112.8 ± 3.3 ^a^	116.1 ± 2.2 ^a^	116.1 ± 2.4 ^a^	110.9 ± 1.6 ^a^
Final body weight (%) Day 14	104.4 ± 0.8 ^a^	86.5 ± 2.2 ^b^	96.9 ± 2.3 ^ac^	92.5 ± 2.4 ^bc^	94.3 ± 1.7 ^bc^	96.3 ± 2.1 ^ac^
Average Daily Feed Intake (g/day)	3.33 ± 0.03 ^a^	2.95 ± 0.03 ^b^	3.06 ± 0.03 ^b^	2.92 ± 0.03 ^b^	2.91 ± 0.05 ^b^	2.95 ± 0.02 ^b^
Average Daily Water Intake (g/day)	4.46 ± 0.03 ^a^	4.27 ± 0.03 ^a^	4.91 ± 0.56 ^a^	3.91 ± 0.47 ^a^	3.98 ± 0.06 ^a^	3.73 ± 0.04 ^a^

Results are expressed as mean ± SE (*n* = 8). Different lowercase superscript letters in the same column indicate significant differences (*p* < 0.05).

## Data Availability

The original contributions presented in this study are included in the article/Supplementary Material. Further inquiries can be directed to the corresponding authors.

## Supplementary Materials

The following supporting information can be downloaded at: https://www.mdpi.com/article/10.3390/cimb48010032/s1.

## Abbreviations

The following abbreviations are used in this manuscript:

IBD	Inflammatory bowel disease
CD	Crohn’s disease
UC	Ulcerative colitis
PME	Prebiotic mixture extract
HI-FBS	Heat-inactivated fetal bovine serum
ABTS	2,2′-azino-bis (3-ethylbenzothiazoline-6-sulfonic acid)
DPPH	2,2-diphenyl-1-picrylhydrazyl
AC	Ascorbic acid
MTT	3-(4,5-dimethylthiazol-2-yl)-2,5-diphenyl tetrazolium bromide
DMSO	Dimethyl sulfoxide
LPS	*Escherichia coli* strain 0111:B4
qRT-PCR	quantitative real-time polymerase chain reaction
DSS	Dextran sodium sulfate
DAI	Disease Activity Index
FD-4	Fluorescein isothiocyanate (FITC)-dextran
KHBB	Krebs–Henseleit bicarbonate buffer
H&E	Hematoxylin and Eosin
TNF	Tumor necrosis factor α
IL-1β	Interleukin 1 beta
NF-κB1	Nuclear factor kappa B subunit 1
TJP1	Tight junction protein 1 (Zonula occludens-1)
TJP2	Tight junction protein 2 (Zonula occludens-2)
OCLN	Occludin
CLDN1	Claudin-1
CLDN4	Claudin-4
GAPDH	Glyceraldehyde-3-phosphate dehydrogenase
IL-6	Interleukin 6
TC	Total cholesterol
TG	Triglyceride
GLC	Glucose
BUN	Blood urea nitrogen
AST	Aspartate aminotransferase
ALT	Alanine aminotransferase
PGE_2_	Prostaglandin E2
COX-2	Cyclooxygenase-2
ASVs	Amplicon sequence variants
PCoA	Principal coordinated analysis
PERMANOVA	Permutational multivariate analysis of variance
LDA	Linear discriminant analysis
