# Supplementary material for: Combined Prebiotic Extract of Mung Bean, Red Bean, and Fennel Improves Intestinal Barrier Integrity in HT-29 Cells and DSS-Induced Colitis via Gut Microbiota Alteration
Source: Curr Issues Mol Biol. 2025 Dec 26;48(1):32. doi: 10.3390/cimb48010032 (PMC12839834; doi:10.3390/cimb48010032)

Figure S1. HPLC chromatograms of (a) vitexin standard and (b) the prebiotic mixture extract (PME) showing the vitexin peak.

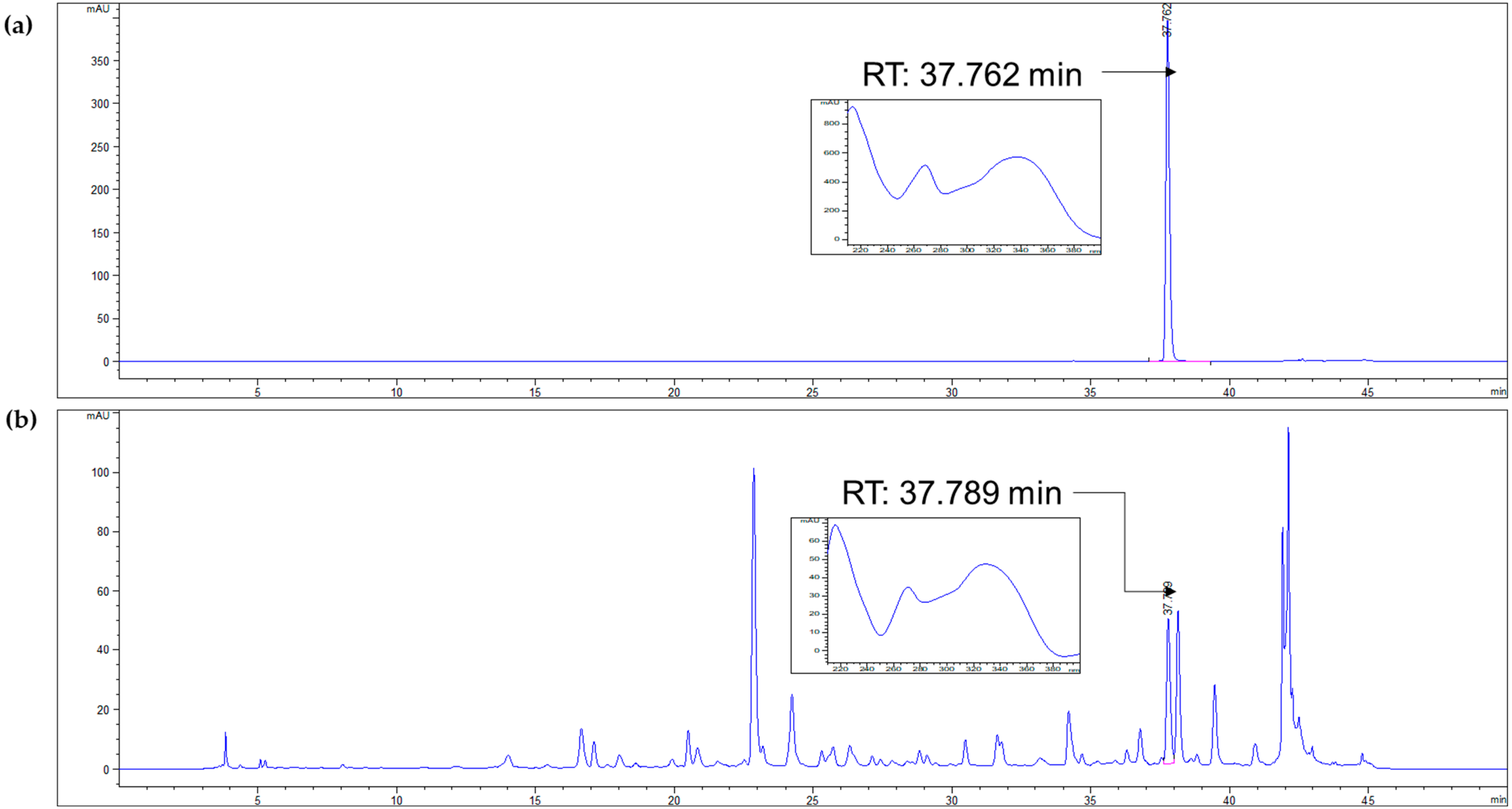

Supplement: Supplementary file 1 [file cimb-48-00032-s001.zip › Figure S1.pdf]
