# Supplementary material for: Combined Prebiotic Extract of Mung Bean, Red Bean, and Fennel Improves Intestinal Barrier Integrity in HT-29 Cells and DSS-Induced Colitis via Gut Microbiota Alteration
Source: Curr Issues Mol Biol. 2025 Dec 26;48(1):32. doi: 10.3390/cimb48010032 (PMC12839834; doi:10.3390/cimb48010032)
Supplement: Supplementary file 1 [file cimb-48-00032-s001.zip › Table S1.pdf]

Table S1. Histopathological scores of H&E-stained distal colon tissues in DSS-induced colitis mice treated with prebiotic mixture extract (PME).

Group	G1										G2									
Animal ID	1	2	3	4	5	6	7	8	Sum	Average	1	2	3	4	5	6	7	8	Sum	Average
Large intestine																				
-Inflmmation severity	0	0	0	0	0	0	0	0	0	0	12	4	3	4	8	8	2	0	41	5.13
-Inflmmation severity score	0	0	0	0	0	0	0	0	0	0	3	1	3	1	2	2	2	0	14	1.75
*Percent involvement(%)	0	0	0	0	0	0	0	0	0	0	4	4	1	4	4	4	1	0	22	2.75
-Inflammation extent	0	0	0	0	0	0	0	0	0	0	4	8	2	4	4	8	2	0	32	4.00
-Inflammation extent score	0	0	0	0	0	0	0	0	0	0	1	2	2	1	1	2	2	0	11	1.38
*Percent involvement(%)	0	0	0	0	0	0	0	0	0	0	4	4	1	4	4	4	1	0	22	2.75
-Crypt damage	0	0	0	0	0	0	0	0	0	0	16	16	4	4	16	16	3	0	75	9.38
*Crypt damage score	0	0	0	0	0	0	0	0	0	0	4	4	4	1	4	4	3	0	24	3.00
*Percent involvement(%)	0	0	0	0	0	0	0	0	0	0	4	4	1	4	4	4	1	0	22	2.75
Individual total	0	0	0	0	0	0	0	0	0	0	32	28	9	12	28	32	7	0	148	18.50
Group total average										0										18.50

Group	G3										G4									
Animal ID	1	2	3	4	5	6	7	8	Sum	Average	1	2	3	4	5	6	7	8	Sum	Average
Large intestine																				
-Inflmmation severity	0	2	0	2	0	0	1	1	6	0.75	0	1	4	0	0	0	0	1	6	0.75
-Inflmmation severity score	0	2	0	1	0	0	1	1	5	0.63	0	1	2	0	0	0	0	1	4	0.50
*Percent involvement(%)	0	1	0	2	0	0	1	1	5	0.63	0	1	2	0	0	0	0	1	4	0.50
-Inflammation extent	0	1	0	2	0	0	1	1	5	0.63	0	2	2	0	0	0	0	1	5	0.63
-Inflammation extent score	0	1	0	1	0	0	1	1	4	0.50	0	2	1	0	0	0	0	1	4	0.50
*Percent involvement(%)	0	1	0	2	0	0	1	1	5	0.63	0	1	2	0	0	0	0	1	4	0.50
-Crypt damage	0	4	0	8	0	0	3	16	31	3.88	4	16	3	0	0	0	0	4	27	3.38
*Crypt damage score	0	1	0	2	0	0	3	4	10	1.25	2	4	3	0	0	0	0	1	10	1.25
*Percent involvement(%)	0	4	0	4	0	0	1	4	13	1.63	2	4	1	0	0	0	0	4	11	1.38
Individual total	0	7	0	12	0	0	5	18	42	5.25	4	19	9	0	0	0	0	6	38	4.75
Group total average										5.25										4.75

Group	G5										G6									
Animal ID	1	2	3	4	5	6	7	8	Sum	Average	1	2	3	4	5	6	7	8	Sum	Average
Large intestine																				
-Inflmmation severity	0	0	1	1	0	0	2	0	4	0.50	0	2	1	0	0	0	2	0	5	0.63
-Inflmmation severity score	0	0	1	1	0	0	2	0	4	0.50	0	1	3	0	1	0	1	0	6	0.75
*Percent involvement(%)	0	0	1	1	0	0	1	0	3	0.38	0	1	3	0	1	0	1	0	6	0.75
-Inflammation extent	0	0	1	1	0	0	1	0	3	0.38	0	0	3	0	1	0	1	0	5	0.63
-Inflammation extent score	0	0	1	1	0	0	1	0	3	0.38	0	0	0	0	1	0	1	0	2	0.25
*Percent involvement(%)	0	0	1	1	0	0	1	0	3	0.38	0	2	0	0	1	0	0	0	3	0.38
-Crypt damage	0	0	3	4	0	1	16	0	24	3.00	0	1	0	0	1	0	0	0	2	0.25
*Crypt damage score	0	0	3	1	0	1	4	0	9	1.13	1	1	0	0	3	0	1	0	6	0.75
*Percent involvement(%)	0	0	1	4	0	1	4	0	10	1.25	1	1	1	0	1	0	1	1	6	0.75
Individual total	0	0	5	6	0	1	19	0	31	3.88	0	3	4	0	2	0	3	0	12	1.50
Group total average										3.88										1.50
